# Uromodulin as a genetically anchored biomarker stratifies time to valve intervention in aortic regurgitation

**DOI:** 10.3389/fcvm.2026.1839906

**Published:** 2026-05-14

**Authors:** Emily Ghanbari, Anastasiia Diagel, Elisabeth Strässler, Anna Sannino, Nicolle Kränkel, Markus Reinthaler, Ulf Landmesser, Ursula Rauch-Kröhnert, Zhifen Chen

**Affiliations:** 1 Department of Cardiology, Angiology and Intensive Care, German Heart Center of Charité, Campus Benjamin Franklin, Berlin, Germany; 2 German Center for Cardiovascular Research (DZHK), Partner Site Berlin, Berlin, Germany; 3 Institute of Active Polymers and Berlin-Brandenburg Center for Regenerative Therapies, Helmholtz-Zentrum Hereon, Teltow, Germany; 4 Department of Cardiology, German Heart Center Munich, Technical University Munich, Munich, Germany; 5 German Center for Cardiovascular Research (DZHK), Partner Site Munich, Munich, Germany; 6 Friede Springer Cardiovascular Prevention Center at Charité, Berlin, Germany

**Keywords:** aortic regurgitation (AR), aortic valve replacement (AVR), biomarker, risk stratification, UMOD gene

## Abstract

**Background:**

Aortic regurgitation (AR) frequently remains asymptomatic for prolonged periods, with guideline recommendations for intervention largely guided by imaging-based assessment of left ventricular remodeling. While renal dysfunction has been linked to adverse outcomes in AR, prior studies have predominantly focused on advanced disease stages and post-interventional outcomes, with limited data on pre-interventional progression, offering limited insight into pre-interventional disease progression or biologically informed approaches to risk stratification.

**Methods:**

We investigated renal biomarkers in individuals with AR from the UK Biobank (*n* = 2,493) and assessed associations with time to aortic valve replacement (AVR) using Cox proportional hazards models. Associations with cardiovascular imaging and hemodynamic phenotypes were examined via logistic regression. To place clinical findings in a biological context, we performed genome-wide colocalization analyses between AR phenotypes, blood pressure, and renal traits, followed by regulatory annotation, single-cell RNA sequencing (Kidney Cell Atlas), and phenome-wide association analyses.

**Results:**

Among patients with AR, higher circulating UMOD levels were independently associated with a lower risk of subsequent AVR (adjusted HR per SD 0.57, 95% CI 0.34–0.98; *p* = 0.041), whereas conventional renal markers (creatinine, urea, microalbumin) showed no association. UMOD levels were strongly associated with lower arterial stiffness (*β* = −5.32 ± 0.27; *p* < 0.001 and lower systolic blood pressure (*β* = −0.91 ± 0.13; *p* < 0.001), both associated with disease progression, but not with left ventricular volumes. Genome-wide colocalization identified a shared genetic signal at the UMOD locus linking AR, blood pressure, and kidney function. Regulatory annotation and single-cell data localized UMOD expression to the thick ascending limb of Henle's loop, supporting a tubular-specific mechanism. Phenome-wide analyses further implicated UMOD variants in renal tubular and hemodynamic traits.

**Conclusions:**

These findings identify uromodulin as a genetically anchored, kidney-specific biomarker associated with pre-interventional progression and timing of valve intervention in AR. Our study shifts the renal–AR paradigm from post-operative risk modification toward a mechanistically grounded model of tubular-driven disease progression, with implications for earlier risk stratification and surgical referral.

## Graphical Abstract

**Figure:**
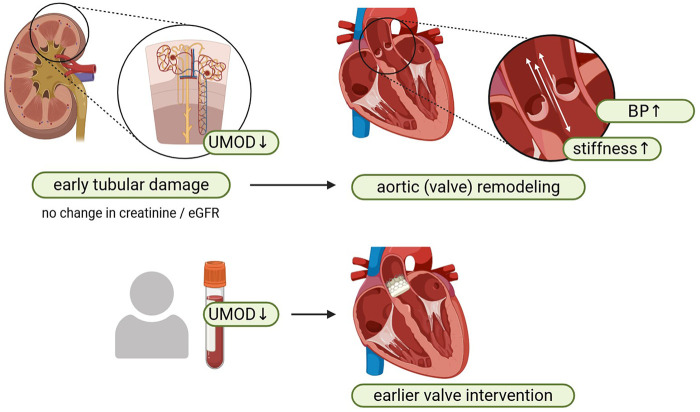

## Introduction

Aortic regurgitation (AR) often remains asymptomatic despite substantial volume overload (1), with current intervention thresholds based on symptom onset or left ventricular (LV) dysfunction due to pressure overload (2–5). Accumulating evidence indicates that adverse outcomes may already be present by the time these criteria are met (6), highlighting the limitations of imaging-only decision frameworks and prompting interest in earlier and biologically informed risk stratification (4, 6, 7).

Prior work on renal dysfunction in AR has largely been confined to its effect on post-interventional survival and peri-procedural outcomes, with minimal insight into early disease stages, pre-interventional progression, or kidney-mediated mechanisms of valvular remodeling (8–12). While these data establish chronic kidney disease as a prognostic modifier, they provide little insight into early or pre-interventional disease progression and do not inform the timing of valve intervention. As a result, both a mechanistic explanation for the renal-AR association and biologically grounded biomarkers to guide intervention timing are lacking.

Renal biomarkers capture distinct aspects of kidney biology, ranging from glomerular filtration and tubular injury to tubular secretory function. Uromodulin (UMOD) is a kidney-specific, genetically anchored marker of subclinical renal tubular biology beyond conventional kidney function measures. It remains unclear whether kidney-specific tubular biology plays a causal role in aortic regurgitation progression and clinical outcomes independent of overt renal dysfunction.

We integrated renal biomarkers with genetic and functional data to identify kidney-specific predictors of intervention timing in AR.

## Methods

### Clinical validation of renal biomarkers and risk of aortic valve intervention

UK Biobank is a large prospective population-based cohort study of approximately 500,000 participants from across the United Kingdom, with linked demographic, clinical, biomarker, imaging, hospital admission, procedural, and mortality data (13). For outcome analyses, we identified 2,493 participants with non-rheumatic aortic regurgitation based on inpatient ICD-10 coding (I35.1). Aortic valve interventions were ascertained from linked hospital procedure records using OPCS-4 surgical codes for valve replacement (K26–K35).

Baseline renal biomarkers measured at UK Biobank assessment included circulating uromodulin, serum albumin, urea, creatinine, and urinary microalbumin. Serum uromodulin (UMOD) was quantified using the Olink Explore 3,072 platform (Olink Proteomics, Uppsala, Sweden), a proximity extension assay–based multiplex proteomics platform and expressed as normalized protein expression (NPX) values on a log₂ scale, reflecting relative protein abundance rather than absolute concentrations. Urea and creatinine were measured from serum samples, and urinary microalbumin was assessed from spot urine samples.

Time-to-event analyses evaluated associations between renal biomarkers and time to aortic valve replacement (AVR), with follow-up defined from first recorded AR diagnosis to AVR, death, or end of follow-up. Associations were assessed using Cox proportional hazards models, applying univariable and age- and sex-adjusted models. Hazard ratios are reported per one standard deviation increase in biomarker level with 95% confidence intervals. Kaplan–Meier curves stratified by biomarker tertiles were generated for descriptive purposes only. Potential non-linearity in the association between UMOD and outcome was assessed by modeling UMOD using restricted cubic splines with 3 degrees of freedom in adjusted Cox proportional hazards models. Non-linearity was evaluated by comparing the spline model with the corresponding linear model using a likelihood ratio test (14–16).

Linear regression models were used to assess the association between circulating UMOD levels and cardiovascular phenotype. Cardiovascular parameters included pulse wave arterial stiffness index measured by finger photoplethysmography, automated systolic blood pressure, and cardiac structure and function assessed by standardized cardiac magnetic resonance imaging, including left ventricular volumes, mass, and cardiac output. All models were adjusted for age, sex, body mass index (BMI), and eGFR (to account for renal function).

Prior to analysis, variables were screened for missingness, coding consistency, and implausible values based on UK Biobank field definitions. Analyses were performed using complete-case analysis, excluding participants with missing covariate data or unavailable laboratory measurements, including UMOD and other renal biomarkers. To assess for potential selection bias, baseline characteristics were compared between participants included and excluded from secondary analyses. Continuous variables are presented as mean ± SD or median [IQR], as appropriate, and categorical variables as percentages. Group comparisons were performed using Student's t-test or Wilcoxon rank-sum test for continuous variables and chi-squared or Fisher's exact test for categorical variables. All statistical analyses were conducted using R (version 4.5.2; R Foundation for Statistical Computing, Vienna, Austria).

### Genome-wide colocalization

We performed genome-wide colocalization analyses between aortic regurgitation phenotypes and a range of cardiometabolic traits, including blood pressure and renal function parameters. Analyses were conducted using the coloc R package (v5.1.0) (17). A posterior probability for shared causality (PP.H4) greater than 0.8 was interpreted as strong evidence supporting a shared causal variant between traits.

Data was used from publicly available summary statistics databases from UK Biobank (refer to Supplementary material). Aortic regurgitation fraction (ARF) GWAS was selected as a surrogate marker for evaluating AR based on prior research. ARF was established as a distinct MRI-derived flow phenotype for aortic regurgitation (18). Across 39,745 UK Biobank individuals included, the mean age at the time of phase-contrast MRI was 64.5 ± 7.7 years, and mean ARF was 7.7 ± 6.3%. Previous multivariable analysis identified only ascending aortic aneurysm and male sex as independent predictors of ARF, underscoring its role as a standalone trait suitable for genetic analysis (18).

### Regulatory annotation

We queried RegulomeDB (v2.0) (19) and HaploReg (v4.1) (20) to retrieve information on chromatin state, transcription factor binding, DNase hypersensitivity, and histone modification marks across relevant tissues (e.g., kidney, vascular endothelium). RegulomeDB ranks (1–7) and integrated scores were used to prioritize variants with likely regulatory effects. LD structure among top SNPs (r^2^ > 0.8) was assessed using 1,000 Genomes European reference data to support variant clustering (20).

### Single-cell RNAseq

We obtained single-cell RNA-sequencing data from the Kidney Cell Atlas (21), which provides curated transcriptomic profiles of distinct human kidney cell types and states.

### PheWAS

We queried the Open Targets Genetics platform (22) to explore phenome-wide associations (PheWAS). Trait-level associations were examined across genome-wide association studies (GWAS) to assess the breadth of phenotypic relevance and prioritize traits linked to cardiovascular pathways.

## Results

We analyzed a UK Biobank cohort of patients with AR (*n* = 2,493) to evaluate the association between renal biomarkers, imaging and hemodynamic phenotypes, and subsequent aortic valve intervention to evaluate biomarkers for early risk stratification.

The study cohort comprised predominantly older male participants (mean age 60.6 ± 6.9 years; 74.3% male) with moderate cardiovascular risk profiles. During follow-up, 23.1% underwent aortic valve intervention, with a mean time to intervention of 4.1 ± 6.9 years. Xenograft replacement was the most common procedure (13.5%), followed by prosthetic valve replacement (7.7%). Baseline renal function was largely preserved (mean eGFR 81.4 ± 19.0 mL/min/1.73 m^2^), with wide variability in urinary microalbumin levels. Participants exhibited elevated systolic blood pressure and modest left ventricular end-diastolic volumes at baseline (Table 1).

**Table 1:** Baseline characteristics of participants included in the complete-case analysis and those excluded in the UK biobank AR cohort (included *n* = 213). Continuous variables are presented as mean ± SD or median [IQR], as categorical variables as %. *p*-values were derived using Student's *t*-test or Wilcoxon rank-sum test for continuous variables and chi-squared or Fisher's exact test for categorical variables. UMOD levels are reported as normalized protein expression (NPX) values on a log₂ scale.

Variable	Included	Excluded	*p*-value
Age at recruitment (years)	59.0 ± 6.9	60.0 ± 7.1	0.521
Male (%)	55.4%	56.6%	0.748
BMI (kg/m^2^)	27.9 ± 5.2	28.6 ± 4.5	0.539
Aortic valve intervention (%)	10.3%	9.3%	0.630
K26.2 Xenograft (%)	8.5%	7.6%	0.674
K26.3 Prosthetic (%)	2.3%	1.3%	0.243
K26.4 Replacement NEC (%)	0.0%	0.2%	1.000
K26.1 Allograft (%)	0.0%	0.1%	1.000
K26.8 Other repair (%)	0.5%	0.1%	0.286
Time to valve intervention (years)	7.9 ± 1.9	8.0 ± 1.7	0.402
UMOD (NPX, log₂ scale)	0.0 ± 1.0	-	-
Creatinine (mg/dL)	0.8 ± 0.1	1.0 ± 0.3	0.066
eGFR (mL/min/1.73m^2^)	82.4 ± 30.5	75.6 ± 26.7	0.305
Albumin (g/L)	44.8 ± 2.8	44.8 ± 2.8	0.978
Microalbumin in urine (mg/L)	15.4 [10.9–36.3]	13.2 [8.8–27.8]	0.029
Systolic blood pressure (mmHg)	143.8 ± 18.5	144.9 ± 20.7	0.446
Pulse wave arterial stiffness index	10.1 ± 3.1	10.7 ± 3.0	0.636
LV end diastolic volume (mL)	54.9 ± 9.5	53.4 ± 9.3	0.636

Analyses were performed using complete-case analysis, excluding participants with missing covariate data or unavailable laboratory measurements, including UMOD and other renal biomarkers. Baseline characteristics of patients included in the complete-case analysis and those excluded in the UK Biobank AR cohort showed no clinically meaningful differences. Despite a statistically significant difference in urinary microalbumin (*p* = 0.029), the small effect size (*r* = 0.08) suggests negligible clinical relevance. These findings suggest that exclusion due to missing data is unlikely to have introduced substantial selection bias.

**Figure 1:**
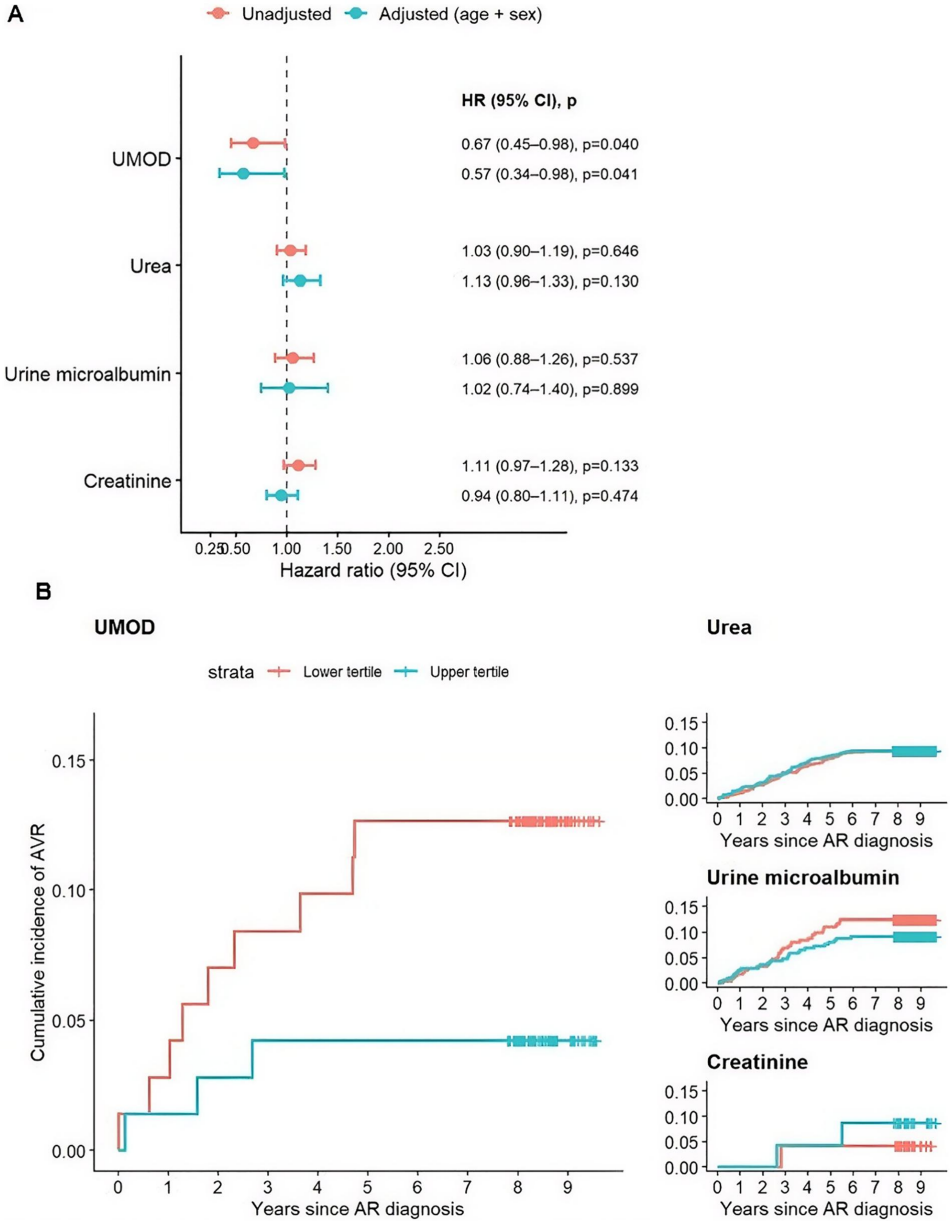
Renal biomarkers and risk of aortic valve intervention. **(A)** Hazard ratios (HRs) with 95% confidence intervals from Cox proportional hazards models evaluating time to aortic valve replacement (AVR) per 1-SD increase in biomarker levels. Results are shown for unadjusted models and models adjusted for age and sex. **(B)** Kaplan–Meier curves for cumulative incidence of AVR stratified by lower versus upper biomarker tertiles for UMOD (left), Urea, urine microalbumin and creatinine. Tick marks indicate censoring.

In Cox proportional hazards analyses, higher circulating UMOD levels were associated with a significantly lower risk of subsequent AVR in both unadjusted models (HR 0.67, 95% CI 0.45–0.98, *p* = 0.040) and after adjustment for age and sex (HR 0.57, 95% CI 0.34–0.98, *p* = 0.041) (Figure 1). Consistent with these findings, Kaplan–Meier analyses demonstrated a clear separation in AVR incidence between lower and upper UMOD tertiles over follow-up. Spline analysis showed no evidence that the relationship was non-linear (*p* = 0.368), indicating that higher UMOD levels were associated with a steadily lower risk of AVR across the observed range.

In contrast, urea, urine microalbumin, and creatinine showed no significant association with AVR risk in either unadjusted or adjusted models, and Kaplan–Meier curves for these markers exhibited minimal separation.

We further aimed to assess the clinical relevance of UMOD in relation to arterial remodeling via MRI-derived parameters. Higher UMOD levels were strongly associated with lower pulse wave arterial stiffness index (*β* = −5.32 ± 0.27, *p* < 0.001) and reduced systolic blood pressure (SBP) (*β* = −0.91 ± 0.13, *p* < 0.001), consistent with a favorable vascular phenotype. No significant associations were detected with left ventricular volumes, mass, or output. These results further show that circulating UMOD levels are linked to hemodynamic load and associated early remodeling indices of the aortic valve.

### *UMOD* locus colocalizes with renal and hemodynamic traits

**Figure 2:**
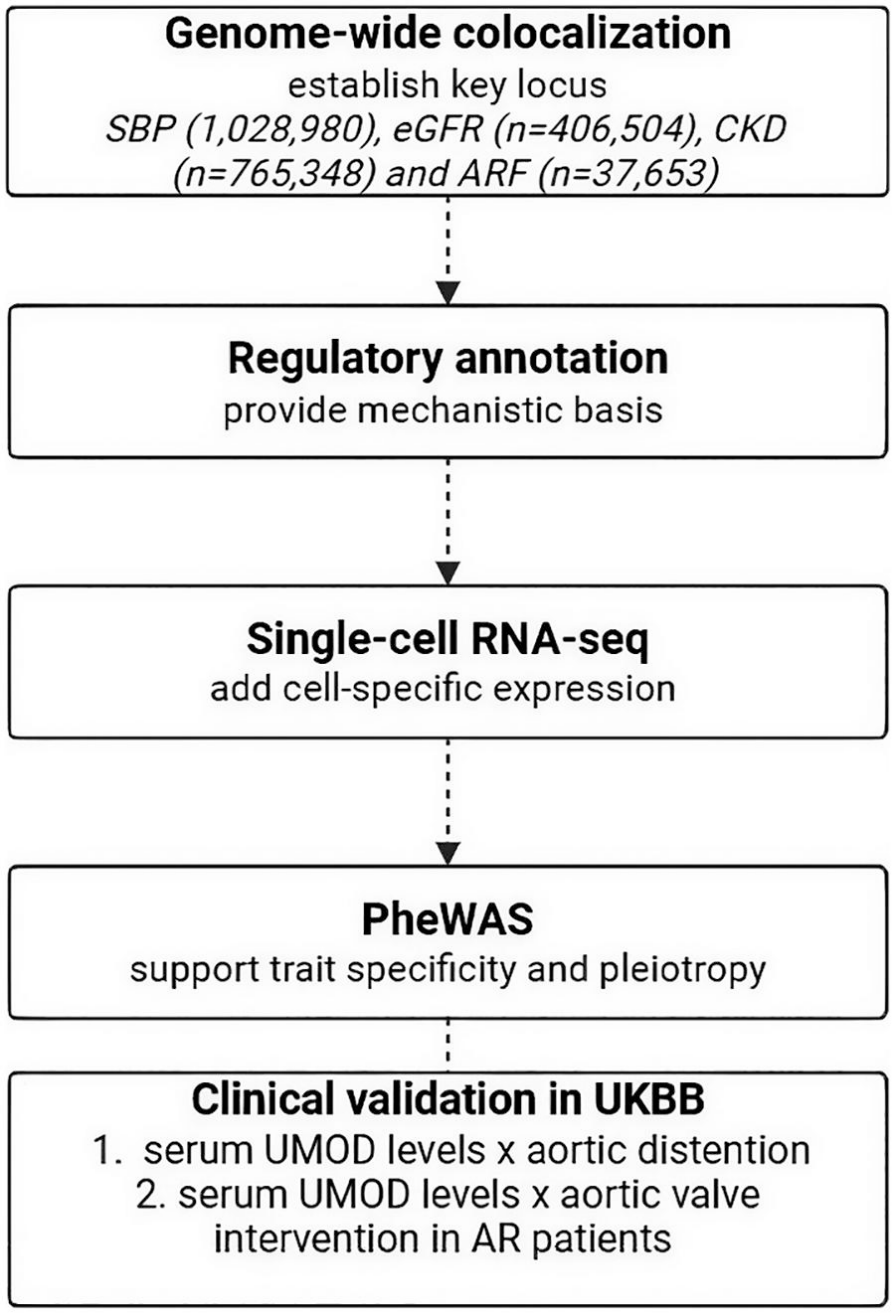
Methods workflow.

**Figure 3:**
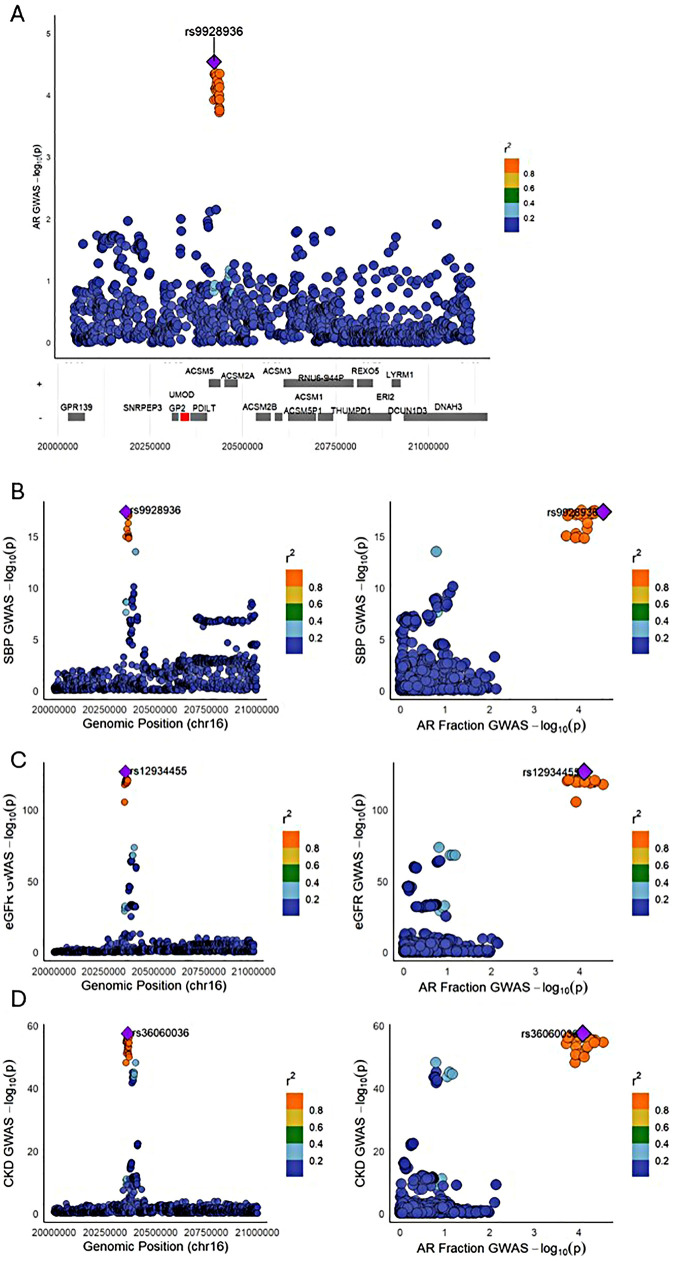
Regional association and colocalization at the UMOD locus on chromosome 16. **(A)** AR GWAS association plot with SNPs colored by LD (r²) to lead SNP rs9928936 (purple diamond). Genes in the region are shown below. **(B-D)** Left panels: GWAS association plots for SBP, eGFR, and CKD, respectively, colored by LD to their lead SNPs. Right panels: Scatterplots showing colocalization of SNP associations between AR fraction and each trait, indicating shared genetic signals.

We conducted genome-wide colocalization analyses to identify loci jointly influencing AR and related traits, including SBP, eGFR, and CKD (Figure 2). A single locus on chromosome 16p12.3, encompassing the UMOD gene, showed strong evidence of a shared genetic architecture with all three traits (PP.H4 = 0.91 for each). Regional association analysis of the AR GWAS signal at this locus revealed a distinct peak centered on rs9928936 (*p* = 2.9 × 10⁻⁵; Figure 3A), with multiple variants in high linkage disequilibrium (r^2^ > 0.8, orange) clustering within the *UMOD* genomic segment. Colocalization plots (Figures 3B–D) show that the association signals for AR and each related trait lie within the same 20 kb window, with concordant LD structure consistent with a single underlying variant set.

### Regulatory annotation supports rs36060036 as likely functional variant

Among significant SNPs at the *UMOD* locus, rs36060036 showed the strongest regulatory potential (Table 2), with evidence of DNase hypersensitivity, enhancer marks, and transcription factor binding (RegulomeDB rank = 4) (Table 2), supporting this variant as a likely shared functional mediator of cardiorenal traits.

**Table 2:** Regulatory annotation of lead SNPs at the UMOD locus colocalized with ARF, BP and kidney traits. Annotation scores were obtained from RegulomeDB and HaploReg. A lower RegulomeDB rank and higher score indicate stronger evidence for regulatory function (e.g., transcription factor binding, DNase hypersensitivity, histone marks). All variants are in strong linkage disequilibrium (LD) (r^2^ > 0.9), supporting a shared causal architecture. LD calculated in EUR population using LDlink.

rsID	Trait	Chr:Pos (hg19)	RegulomeDB Rank	Score	DNase	TFBS	Enhancer Marks	r^2^ with rs36060036
rs36060036	CKD	chr16:20350627	4	0.609	Yes	Yes	Yes (K562)	1.00
rs12934455	eGFR	chr16:20345958	7	0.184	No	No	Weak	0.96
rs9928936	SBP	chr16:20341726	6	0.005	No	No	None	0.93

### *UMOD* expression is localized to thick ascending limb of Henle's loop in kidney

**Figure 4:**
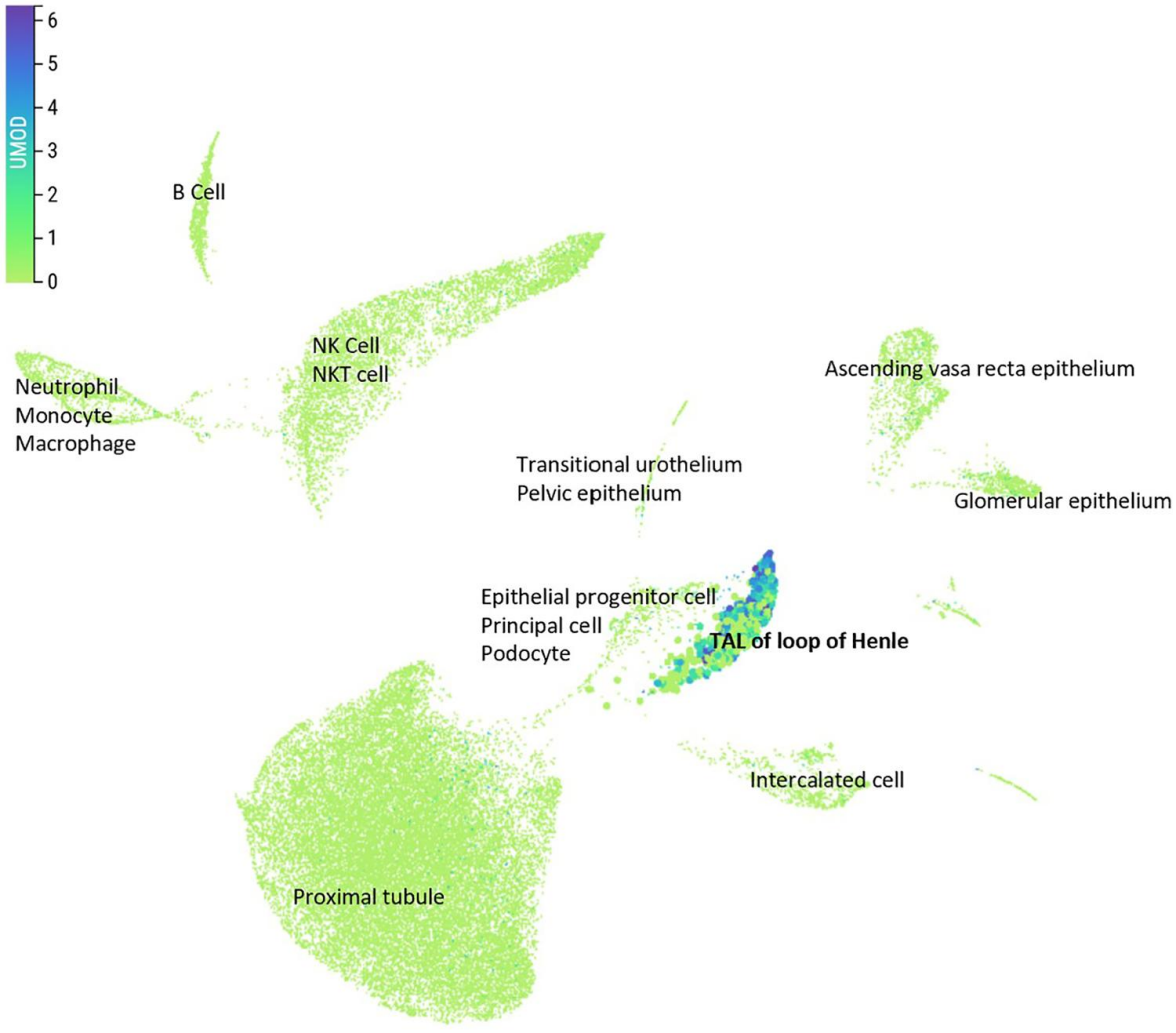
UMOD is selectively expressed in the thick ascending limb of Henle's loop in kidney. *UMOD* expression levels in mature human kidney single-cell RNA-seq data from the Kidney Cell Atlas [21]. Uniform Manifold Approximation and Projection (UMAP) visualization highlights cell type-specific expression across >100,000 cells. *UMOD* expression is confined to epithelial cells of the TAL of Henle's loop, with negligible signal in other nephron segments. Color scale reflects normalized *UMOD* expression. Image obtained and modified from Kidney Cell Atlas [21].

To assess the renal cell-type specificity of *UMOD* expression, we analyzed single-cell transcriptomic data from the Kidney Cell Atlas (21). In over 100,000 mature human kidney cells, *UMOD* expression was highly restricted to the thick ascending limb (TAL) of Henle's loop, with no appreciable expression in glomerular, proximal tubule, or collecting duct cells (Figure 4). This specific expression pattern likely explains no significant cis-eQTLs of the *UMOD* locus in GTEx bulk kidney, in which TAL cells represent a minority. These data support the notion that *UMOD* exerts its biological effects specifically within TAL-associated functions, including sodium handling and tubuloglomerular feedback.

### Phenome-wide associations link UMOD to renal and tubular traits

**Figure 5:**
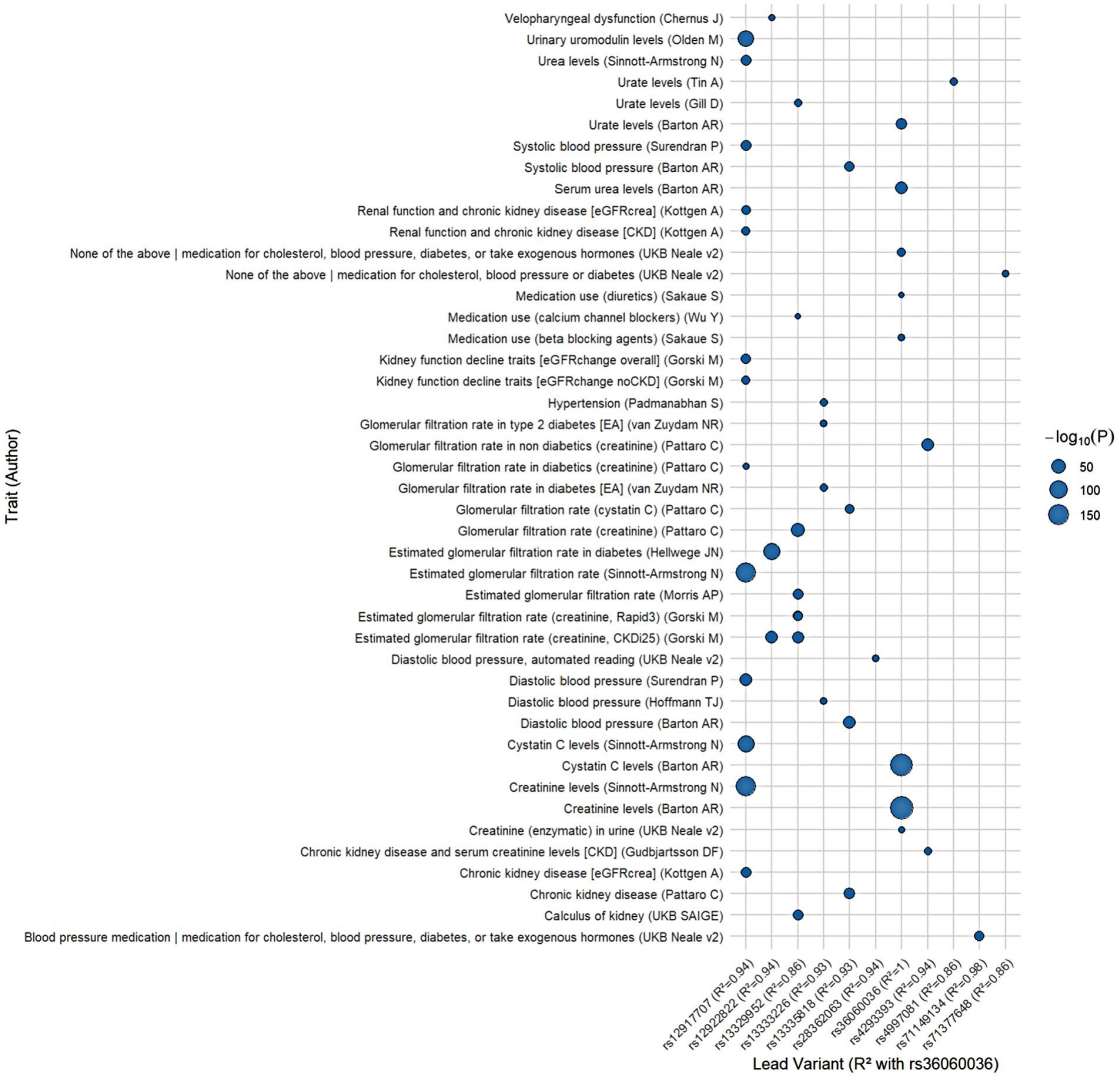
PheWAS plot for variants in high linkage disequilibrium (LD, R2 > 0.8) with rs36060036. Each point represents the association between a trait and a lead variant that shares high LD with rs36060036, annotated by study author. The *x*-axis displays lead variants labeled by rsID and their LD (R2) with rs36060036. The *y*-axis shows the associated trait and publication. Point size indicates the strength of association as −log_10_(*p*-value). Associations span multiple domains, including kidney function, blood pressure regulation, and medication use.

To explore the phenotypic spectrum of variants at the *UMOD* locus, we conducted a PheWAS across all traits significantly associated with variants in strong LD with the lead variant (rs36060036) (R^2^ > 0.8). As shown in Figure 5, variants of the *UMOD* locus were further associated with a spectrum of traits, showing biological relevance to cardiovascular and renal physiology.

Notably, rs36060036 or its LD SNPs showed strong associations with GFR, CKD and serum levels of uromodulin, urea, urate, and creatinine. For example, rs36060036 itself was genome-wide significantly associated with urinary uromodulin levels (*p* = 3 × 10⁻⁹), serum urate (*p* = 4.2 × 10⁻^2^⁵), and serum urea (*p* = 7.7 × 10⁻^3^⁵), consistent with its role in tubular function.

Variants in high LD are also associated with blood pressure traits, including systolic and diastolic blood pressure (e.g., rs9928936, R^2^ = 0.26), and with medication use, including beta blockers and diuretics. These findings support the hypothesis that the *UMOD* locus harbors regulatory variants with pleiotropic effects across renal excretory function and hemodynamic regulation, thereby linking tubular handling with cardiovascular risk.

## Discussion

### Summary of findings

In this study, we identify uromodulin (UMOD) as a genetically anchored, kidney-specific marker associated with time until valve intervention in AR. Integrating clinical outcomes, cardiovascular imaging, and genetic analyses, we demonstrate that lower circulating UMOD levels are associated with earlier need for valve replacement in patients with preserved glomerular function. Genome-wide colocalization and regulatory annotation implicate a shared *UMOD* locus linking AR, blood pressure, and renal traits, while single-cell data localize *UMOD* expression to the thick ascending limb of Henle's loop. Together, these findings support a model in which subclinical tubular dysfunction contributes to hemodynamic load and valvular remodeling prior to overt renal impairment via traditional renal markers.

### Implications of UMOD for early risk stratification in aortic regurgitation

Current guideline recommendations for AR rely almost exclusively on imaging-based thresholds and provide no biomarker-based support for timing of intervention (3, 4, 6). Prior work on renal dysfunction in AR has largely focused on overall clinical outcomes and late-stage disease (8, 11, 12). Chronic kidney disease is a well-established modifier of prognosis in aortic valve disease, being associated with higher rates of adverse cardiovascular events and heart failure hospitalization (23), as well as markedly worse long-term survival after both surgical and transcatheter valve replacement; particularly among patients with advanced renal impairment (8, 11, 12). However, this evidence is largely derived from late-stage disease and post-interventional outcomes and does not address whether renal biology contributes to pre-interventional progression or informs when intervention should occur. Moreover, renal dysfunction has typically been defined using glomerular filtration–based measures, limiting insight into kidney-specific mechanisms that may actively drive AR progression (10). As a result, current frameworks lack both a mechanistic explanation for the renal–AR association and a biomarker to inform surgical timing.

In this context, UMOD provides a biologically grounded alternative. As shown in our study, UMOD is a kidney-specific tubular protein with strong genetic anchoring, UMOD enables interrogation of subclinical renal biology as a determinant of AR progression rather than merely a modifier of post-operative risk. Reduced UMOD levels were associated with earlier valve intervention despite preserved creatinine and eGFR, supporting its potential role in identifying patients in which AVR becomes necessary earlier. Our findings extend recent efforts to translate omics discoveries into clinically actionable biomarkers (24) to valvular heart disease, where early risk stratification remains a major unmet need (3, 5).

### Renal tubular dysfunction and valvular remodeling

UMOD is central to tubular integrity, sodium handling, and blood pressure regulation and has been robustly linked to renal and vascular physiology (25, 26). Genetic variation at the *UMOD* locus influences blood pressure and kidney function through enhanced tubular sodium reabsorption via NKCC2 and NCC activation (26–28), but its relevance to valvular disease progression has not previously been examined.

Our findings suggest that *UMOD*-associated tubular dysfunction may manifest as impaired volume handling and increased arterial stiffness, contributing to chronic hemodynamic load on the aorta and aortic valve. This complements Mendelian randomization studies linking UMOD to cardiovascular outcomes primarily through blood pressure (28) and supports a broader role for renal tubular biology in shaping hemodynamic stress relevant to valvular remodeling.

### UMOD as an early marker of AR progression

Unlike creatinine or eGFR, which primarily reflect glomerular function, circulating UMOD has been established as an analytically stable marker (29–31) that captures tubular integrity and inversely correlates with tubular fibrosis (26). In this study, UMOD was assessed as a circulating biomarker using Olink NPX values, enabling standardized relative quantification across individuals. Accordingly, our analysis was designed to evaluate biological stratification rather than to define clinically applicable concentration thresholds. In contrast to urinary biomarkers, where creatinine indexing is commonly used to account for urine dilution, circulating UMOD reflects systemic protein levels, consistent with this, prior work has shown that the creatinine-serum uromodulin ratio does not provide additional value beyond serum UMOD alone (29, 32).

In our study, circulating UMOD was associated with arterial stiffness and the time until valve intervention in a cohort with largely preserved kidney function. This distinction is particularly relevant in AR, where compensatory mechanisms may delay symptom onset despite ongoing maladaptive remodeling (1). Consistent with this, prior meta-analyses have demonstrated that even mild renal impairment is associated with increased cardiovascular mortality and that normal creatinine or eGFR values do not exclude cardiovascular risk (33, 34). Together, these findings provide combined genetic and clinical evidence linking tubular dysfunction to AR progression and support UMOD as a candidate biomarker for early risk stratification.

### Limitations

Several limitations warrant consideration. Clinical validation was based on a clinically defined UK Biobank cohort, with AR identified via inpatient ICD-10 coding, reflecting clinical documentation rather than true disease onset and introducing potential left truncation bias. In addition, ICD-10-based case identification lacks baseline severity granularity, and heterogeneity in disease stage at cohort entry may have influenced time-to-AVR estimates.

The absence of a cis-eQTL for UMOD in bulk kidney tissue likely reflects its restricted expression to the thick ascending limb, which we addressed using single-cell transcriptomic data (21). As circulating UMOD was quantified using relative NPX values, direct translation to absolute clinical concentrations is not possible within the present study, and validation against clinically established quantitative assays such as ELISA or other immunoassays will be required for clinical implementation. Although circulating UMOD appears analytically stable, residual short-term biological variability, including potential effects of sustained physiological exposures, cannot be excluded.

External replication and further mechanistic studies are needed to validate and extend the findings of our study.

## Conclusion

In summary, our findings reposition renal involvement in AR from a late-stage comorbidity to an early, mechanistically relevant contributor to disease progression. By integrating genetic, molecular, imaging, and clinical outcome data, we identify UMOD as a genetically anchored, kidney-specific biomarker associated with pre-interventional progression and timing of valve intervention in AR. These results suggest a potential role for UMOD as a genetically anchored biomarker in refining early risk stratification and informing interventional decision-making beyond current imaging-based frameworks.

## Data Availability

The data analyzed in this study is subject to the following licenses/restrictions: Data from the Genotype-Tissue Expression (GTEx) Project and UK Biobank are available to qualified researchers upon application and approval, under data use agreements; individual-level data cannot be publicly shared. Requests to access these datasets should be directed to Access to data from the Genotype-Tissue Expression (GTEx) Project can be requested via dbGaP (https://dbgap.ncbi.nlm.nih.gov/). Access to the UK Biobank dataset can be requested through the Access Management System (https://www.ukbiobank.ac.uk/enable-your-research/apply-for-access).
